# Supplementary material for: Patterns of Intron Gain and Loss in Fungi
Source: PLoS Biol. 2004 Nov 30;2(12):e422. doi: 10.1371/journal.pbio.0020422 (PMC532390; doi:10.1371/journal.pbio.0020422)
Supplement: Table S1 — Also available at http://genes.mit.edu/NielsenEtAl/. (4.3 MB ZIP). [file pbio.0020422.st001.zip › NielsenEtAl/html/1351.html]

AN6368.1.NCU08195.1.MG09080.1.FG06576.1


```
 CLUSTAL W (1.82) Multiple Sequence Alignments - Introns Inserted


Sequence 1: NCU08195.1	647 aa
Sequence 2: MG09080.1	639 aa
Sequence 3: FG06576.1	635 aa
Sequence 4: AN6368.1	650 aa
Alignment Length: 666 aa
Number Identitical Residues: 316 aa
Alignment Score (without introns) 15688


MG09080.1 	-MPSHASIDQLATLAGDLSLS--EGSDRFPNFYPESNPLDVYRAHLTNILQPITGVDPKI
NCU08195.1	MTAVAAPIDQLVKQVDGLNLD--AVKAKFPDTYPDLNLMDLYRAHLANVLSEVTGVDTAI
FG06576.1 	---MATDANALADQLQELGID--KVES-YPNCHPDTNPVDIYRSHITSLLAPITGVDPSI
AN6368.1  	----MASTDSLPAAVESLSLQTTSETSKFPNSYPSLNPVDVYREHISEHLSAITGIEAEK
          	     :  : *      *.:.::     :*: :*. * :*:** *::. *  :**::.  

MG09080.1 	IYPAVQWTSSLDKGDLVVA~APALRVKGKKPDELVKEWSEK0FPTDDPLFEKPVVTG---
NCU08195.1	IYPNLGWTSGLDKGDIALP~VPSLRVKGKKPDELAQEWAAK0FPDTDPLFTKPTVSQ---
FG06576.1 	IYPAIQWTQTLDKGDAILA~VPALRVKGKKPGELAEEWASK0FPES-PLIEKPTASG---
AN6368.1  	IYPRLQFTSTLDKGDLLLP0VPSLQIKGKSPADLSKELAEK~FPES-DLIHPPIAGGPGG
          	*** : :*. *****  :. .*:*::***.* :* :* : * **    *:  * .  ...

MG09080.1 	YFLQFWFKPDPLTRNLIPLIRELGDDYGNNKRLGLKDPQDPSKGNKKIIVEFSSPNIAKP
NCU08195.1	IHLSFFVKASPLVNAVIPMVQHKGAEYGFNKADGLKDPKDPSKGQKRIIVEFSSPNIAKP
FG06576.1 	PFISFFFKADKLTNMLLPTIRKQASSYGKSPYNGLRDPSDPNSGKKRMIVEFSSPNIAKP
AN6368.1  	THLQFFFKEKPLAQRVIGQIFKEKAAYGSNGNQGLRDPKDPSKGRKKIIVEFSSPNIAKP
          	 .:.*:.* . *.. ::  : .    ** .   **:**.**..*.*::************

MG09080.1 	FHAGHLRSTIIGGFLANLYKGAGWDVQRINYLGDWGKQYGLLALAFERYGDEEALKKDPI
NCU08195.1	FHAGHLRSTIIGGFLSNLYAGAGWDVIRINYLGDWGKQYGLLALAYEKYGDEEALQKDPI
FG06576.1 	FHAGHLRSTIIGGFIASLHESAGWEVTRLNYLGDWGKQYGLLALAWEKWGDEEALKADPI
AN6368.1  	FHAGHLRSTIIGGFLANLYTVMGWDVIKMNYLGDWGKQYGLLANGYKKFGDEQKLLQDPI
          	**************::.*:   **:* ::************** .::::***: *  ***

MG09080.1 	NHLFQLYVKINSEMSEE-------KEQIESKKKAGEDVTQLEQK-------SLDEQARAY
NCU08195.1	NHLFQLYVRINTEMTAE-------KEELTKRKEAGEDVSALEAN-------SLDEQARRY
FG06576.1 	NHLFNLYVRINSDMSDE-------KSKIKEKQDAGEDVSALEAD-------SLDEQARKY
AN6368.1  	NHLFEVYVKINNIVSEQEGPIKELKEQIKAKKEKNEDVSKLEQELAKLVDVSEDENARRY
          	****::**:**. :: :... .. *.::  ::. .***: ** . :.  . * **:** *

MG09080.1 	FKKMVDGDEAAVAQWRRFRDLSIERYKKTYARLNIWFDEYSGESQVTEESMAKSAKTIAD
NCU08195.1	FKKMTDRDADALAMWKRFRDLSIVRYKDTYARLNIHFDEYSGESQVSEEAMNKVGNLMEE
FG06576.1 	FRRMTDGDKEAVDLWRRFRDLSIVRYKKTYARLNIEYDEYCGESQVPEEDMKKAAEILAE
AN6368.1  	FKSMEDGDPDALSLWRRFRDLSIKKYRETYARLNIDYDVYSGESQIKQESMSKANEILQS
          	*: * * *  *:  *:******* :*:.******* :* *.****: :* * *  : : .

MG09080.1 	KGISKEDQGAVIIDFSEHVPGKEGKRLERPIIRKRDGTALYLTRDISELLGRHERYNFDK
NCU08195.1	KKICKEDKGAQLIDFSELVPGKEGKRLEKPLVRKRDGTALYLTRDISELLGRHEKYNFDK
FG06576.1 	KGLTEDANGATLIDFSKHVAGKPGKSLEKVILRKKDGTALYLTRDISELLNRQEKYKFDK
AN6368.1  	TGVSENSDGATIVDFPKHG----AKKLGKAVIVRKDGTPLYLTRDIGAIFERDEAYHFDK
          	. : :: .** ::**.:      .* * : :: ::***.*******. :: *.* *:***

MG09080.1 	MIYVVASQQDLHLKQLFKIVELMGHKEIADKCQHINFGMVLGMSTRKGTVKFLDDILRDV
NCU08195.1	MIYVVASAQDLHLRQLFKIIELLGYKDIADKCQHINFGLVLGMSTRKGTVKFLDDILRDV
FG06576.1 	MIYVIASQQDLHLKQLFKTIELMGHEDIVKKCQHINFGMVLGMSTRKGTVKFLDDILRDV
AN6368.1  	MIYVVAAQQDLHLAQLFKIVELMGFKDLASRCQHINFGMVRGMSTRKGTVKFLDDILRDV
          	****:*: ***** **** :**:*.:::..:*******:* *******************

MG09080.1 	GDKMHEVMRKNDDKYSQVENPEAVADILGISSVMVQDMTGKR2INNYTFDMDAMTSFEGD
NCU08195.1	ADKMHEVMKKNENKYSQIEDPLATADVLGISSVMVQDMSGKR2INNYTFNMETMTSFEGD
FG06576.1 	AEKMHEVMRKNETKYSQVENPEAVADILGISSVMVQDMSGKR2INNYKFDMDVMTSFEGD
AN6368.1  	ADKMHETMKKNEEKYAQIENPVETADILGITSVMVQDMSGRR2INGYDFNLDAMTSFEGD
          	.:****.*:**: **:*:*:*  .**:***:*******:*:* **.* *:::.*******

MG09080.1 	TGPYLQYAHARVCSIARKAGLTPEELATADLSLLQEPHARQLVRMLSQWPDTVQNTYKTL
NCU08195.1	TGPYLQYAHARLNSIRRKAGVSDEELATADLSLLTETSAINLCRHMAGWPDVVKNTLKTL
FG06576.1 	TGPYLQYAHARLCSIRRKVNLSDEQLATADYSLLKEKHATDLVRLLSQWPDVVQNTLKTL
AN6368.1  	TGPYLQYAHSRLCSILRKAEANVEELPSANLDLLTESHAVNLVRLLSSWHDILMMTNKTL
          	*********:*: ** **.  . *:*.:*: .** *  * :* * :: * * :  * ***

MG09080.1 	EPTTVLTYLFRMSHALSQSYDHLRIVGSEKELMKARMALYDATRIVLRNGMILLGLNPVE
NCU08195.1	EPTTVLTYLFKLTHALSSSYDQLQVVGSEPELMKARLALYTAVQTVLANGMRLIGLSPVD
FG06576.1 	EPTTILTYLFKMTHVLSSSYDHLRIVGSEDELQKARMGLYDAARIVLYNGMSLLGLSPVE
AN6368.1  	EPTTILTYLFRLTHMISSSYDVLQVIGSEPELKKARLALYEAARQVLHNGMRILGLSPVS
          	****:*****:::* :*.*** *:::*** ** ***:.** *.: ** *** ::**.**.

MG09080.1 	R~M-------
NCU08195.1	R~YVSPGAPD
FG06576.1 	R2M-------
AN6368.1  	R2M-------
          	*
```
